# Complete chloroplast genome of *Nyctocalos pinnatum*: chloroplast features and phylogenetic relationships within Bignoniaceae

**DOI:** 10.1080/23802359.2022.2081522

**Published:** 2022-06-14

**Authors:** Huan Fan, Jinyue Li, Sven Landrein

**Affiliations:** a Centre for Integrative Conservation, Xishuangbanna Tropical Botanical Garden, Chinese Academy of Sciences, Mengla, China; b Center of Conservation Biology, Core Botanical Gardens, Chinese Academy of Sciences, Mengla, China; c University of Chinese Academy Sciences, Beijing, China; d Center for Gardening and Horticulture, Xishuangbanna Tropical Botanical Garden, Chinese Academy of Sciences, Mengla, China

**Keywords:** Bignoniaceae, *Nyctocalos pinnatum*, phylogeny, chloroplast genome

## Abstract

*Nyctocalos* is a genus of flowering lianas belonging to the family Bignoniaceae, and occurring from South-Central China to Malesia. In this study, we assembled the first complete chloroplast genome of *N. pinnatum.* The total length of the chloroplast genome is 159591 bp, with a GC content of 38.04%, which includes a pair of inverted repeats of 30,480 bp, a small single-copy region of 12,774 bp and a large single-copy region of 85,857 bp. The chloroplast genome contains 135 genes, consisting of 89 protein-coding genes, 38 transfer RNAs, and 8 ribosomal RNAs. We constructed a phylogenomic tree with representative chloroplast genomes from Bignoniaceae. *N. pinnatum* is revealed to be sister to *Oroxylum* in the tribe Oroxyleae, with a high bootstrap support. This is the first chloroplast genome assembled in *Nyctocalos*, and it provides essential information for further ecology and evolutionary studies in this genus and Bignoniaceae.

*Nyctocalos pinnatum* Steenis 1953 is classified in Bignoniaceae, tribe Oroxyleae (Kubitzki 2004). The genus is characterized by its septicidal capsule and Asian distribution. *Nyctocalos* has three species native to South central China to West Malesia (van Steenis 1977; Kubitzki 2004). Two species are found in China, *N. brunfelsiiflorum* and *N. pinnatum*, although the classification and nomenclature of the genus is complex and the two species are often misidentified. *Nyctocalos* flowers have a long, tubular, white reflective and fragrant corollas that only open at night and are said to be pollinated by bats (Corlett 2004). As in other Bignoniaceae its stigmas are sensitive and will shut when touched by a visitor thus avoiding repetitive fertilization (Darwin 1876; Milet-Pinheiro et al. 2009). The Chinese name for this genus means shed light into the night, which reflects well in its ecology and morphology. Other morphological unique characters in *Nyctocalos* as well as *Hieris* are the punctate glands on leaves, calyx, and corolla which attract many ants.

*Nyctocalos* is placed under the tribe Oroxyleae, which includes three other genera, *Millingtonia, Oroxylum, and Hieris*. The phylogeny of the neotropical members of the Bignoniaceae family has been well studied based on two chloroplast markers, *rbcL* and *ndhF* (Spangler and Olmstead 1999), as well as an additional marker, *trnL-F,* and increased sampling (Olmstead et al. 2009), however, the phylogeny of Asian members has not been studied. The placement of tribe Oroxyleae is unresolved and only cladistic analyses of morphological traits is available (Abdel-Hameed 2014).

Here, we assembled and annotated the chloroplast genome of *N. pinnatum* using next-generation sequencing, in order to provide an opportunity for finer scale molecular and taxonomic work in this family and this tribe, such as providing super-barcodes for identification against closely related species (Chen et al. 2020).

We sampled fresh leaves of wild sourced *N. pinnatum,* collected in Yuxi, Yunnan, China, and cultivated in the vine garden of Xishuangbanna Tropical Botanical Garden (Yunnan, China, geospatial coordinates: 21°55′N 101°15′E; altitude: 570 m), Chinese Academy of Sciences. The collection permit was granted by the Center for Gardening and Horticulture of Xishuangbanna Tropical Botanical Garden. The herbarium specimen voucher was deposited in HITBC, Yunnan, China (contact info: Jianwu Li, ljw@xtbg.org.cn) under the voucher number V000030. Total genomic DNA was extracted using Magnetic Plant Genomic DNA Kit (TIANGEN). Ilumina NovaSeq 6000 platform was used for sequencing and pair-end reads with 150 bp each were generated. Libraries preparation and sequencing were performed by Annoroad Gene Technology (Beijing China).

Adaptor sequences were removed from raw data and low quality reads were filtered out. Clean data are used for de novo assembly of the chloroplast genome using GetOrganelle 1.7.5 (Jin et al. 2020). We then mapped the clean reads back to the assembled chloroplast genome to check for assembly errors. As for annotation, we manually compared the automated version generated from CpGAVAS2 (Shi et al. 2019) to *Oroxylum indicum* (NC_049086.1), which is a closely related species using Geneious (v2020.0.5). Finally, the chloroplast DNA sequence and annotations were deposited in GenBank under accession number OK649927.

The chloroplast genome is 159,591 bp in length, including the Large Single Copy (LSC) region (85,857 bp), the Small Single Copy (SSC) region (12,774 bp), and two Inverted Repeats (IR, 30,480 bp). The overall GC content is 38.04%, with the number for LSC and SSC being 36.32% and 33.51%, respectively, which are both lower than in the IR regions (41.4%). The chloroplast genome has 135 genes, consisting of 89 protein-coding, 38 tRNA, and 8 rRNA genes.

To further confirm the placement of *Nyctocalos* in Bignoniaceae, protein-coding sequences from complete chloroplast genomes of 27 Bignoniaceae species that are publicly available including *N. pinnatum* were used to reconstruct the phylogeny of this family. Sequence alignment was performed using MAFFT (Katoh and Standley 2013), topology and supporting rate of the phylogenetic tree were computed using RAxML v8.2.10 (Stamatakis 2014) using the GTR + G substitution model and with 1000 bootstrap. Our tree supports the placement of *Nyctocalos* as sister to *Oroxylum*, which is another genus in Oroxyleae (Figure 1). The backbone relationship between major tribes largely agrees with Olmstead 2009, except that Oroxyleae is a sister group to Crescentiina (bootstrap = 83%) instead of Catalpeae (bootstrap = 48% as reported in Olmstead 2009).

**Figure 1.**
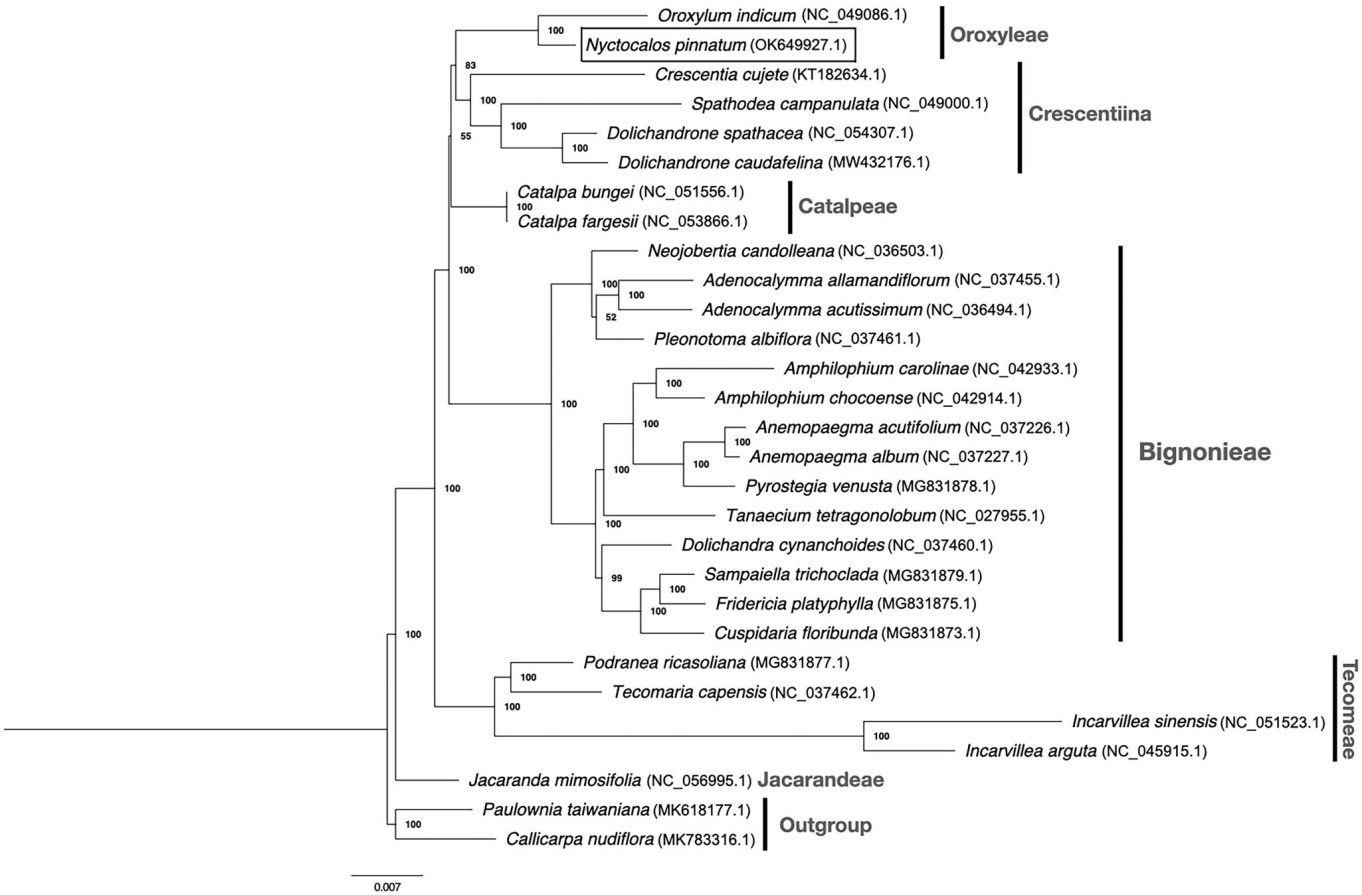
Maximum-likelihood tree of *N. pinnatum* in relation to other 26 species in Bignoniaceae based on protein-coding sequences from complete chloroplast genomes. Bootstrap values are shown next to the nodes.

To conclude, the complete chloroplast genome of *N. pinnatum* is a useful DNA data source for further studies of the evolutionary history of this genus and tribe Oroxyleae and can be used as super-barcodes for identification of this species in ecological studies.

## Author contributions

HF and SL were involved in the conception and design; HF and JL did the analysis; HF drafted the paper; SL revised it critically for intellectual content; all authors approved the final version to be published and agree to be accountable for all aspects of the work.

Acknowledgement

This work was supported by the Core Botanical Gardens under Grant No. Y9ZK011B08. HF was supported by the China Postdoctoral Fund (2019M663597) and International Postdoctoral Exchange Fellowship Program. We als would like to thank Center for Gardening and Horticulture, Xishuangbanna Tropical Botanical Garden, Chinese Academy of Sciences for providing the plant material.

## Data Availability

The genome sequence data that support the findings of this study are openly available in GenBank of NCBI at [https://www.ncbi.nlm.nih.gov] under the accession number OK649927. The associated BioProject, SRA, and Bio-Sample numbers are PRJNA816672, SRR18335031, and SAMN26688270, respectively.
